# Association Between Double Bonuses and Clinical and Administrative Performance in Medicare Advantage

**DOI:** 10.1001/jamahealthforum.2022.3301

**Published:** 2022-09-23

**Authors:** Andrew M. Ryan, Baris Gulseren, John Z. Ayanian, Adam A. Markovitz, David J. Meyers, Erin Fuse Brown

**Affiliations:** 1 Department of Health Management and Policy, School of Public Health, University of Michigan, Ann Arbor; 2 University of Michigan Center for Evaluating Health Reform, Ann Arbor; 3 Department of Internal Medicine, University of Michigan Medical School, Ann Arbor; 4 University of Michigan Institute for Healthcare Policy and Innovation, Ann Arbor; 5 Department of Health Services, Policy & Practice, Brown School of Public Health, Providence, Rhode Island; 6 College of Law, Georgia State University, Atlanta

## Abstract

This cross-sectional study compares double bonuses with clinical and administrative performance in Medicare Advantage facilities.

## Introduction

Nearly 45% of Medicare beneficiaries are enrolled in private Medicare Advantage (MA) plans. In 2012, MA plans became eligible for bonus payments based on 5-star quality ratings. One set of quality measures is related to clinical performance, a second to administrative performance. In double-bonus counties (metropolitan areas with high MA enrollment and low fee-for-service spending), highly rated plans receive bonuses twice as large as those in non–double bonus counties. Spending for double bonuses was approximately $1.5 billion annually from 2012 through 2018.^1^

Associations between double bonuses and clinical and administrative performance are unknown. Plans have more control over administrative operations than clinical performance and may focus efforts on improving administrative performance. However, clinical measures account for approximately 65% of quality ratings, whereas administrative measures account for 35%. In this cross-sectional study, we compared double bonuses with clinical and administrative performance in Medicare Advantage.

## Methods

**Figure 1.**
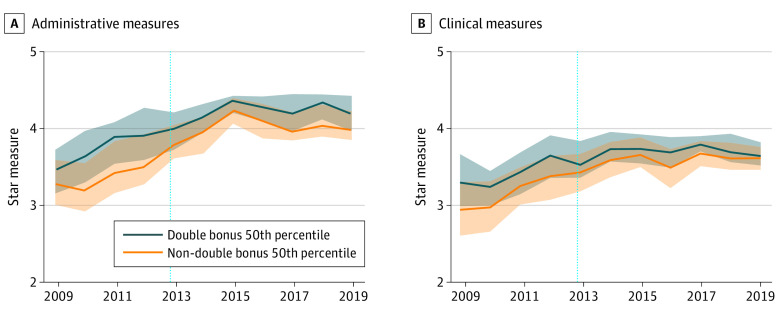
Ratings for Counties That Ever Received Double Bonuses and Counties That Never Received Double Bonuses Dashed vertical line denotes period preceding the start of the Quality Bonus Program. Shaded areas indicate the 25th to 75th percentiles. Further information on the methods used to calculate composite clinical and administrative performance is available in eMethods in the Supplement.

Medicare Advantage plan data for 2009-2019 from the Centers for Medicare & Medicaid Services website were used to define quality performance at the county-plan level^2^; MA Ratebook Files were used to identify double-bonus counties^3^; Medicare Beneficiary Summary Files were used to define beneficiary characteristics. Data were analyzed from January 29, 2022, to May 3, 2022. Race and ethnicity data were identified through beneficiary self-report. For each year, we calculated plans’ composite clinical and administrative performance by taking the mean star rating for all measures in each domain (Figure 1; eMethods in the Supplement). Our analysis adhered to the principles of the STROBE reporting guideline. Our study was deemed exempt by the University of Michigan institutional review board due to the use of publicly available data.

We estimated separate linear regression models for each composite outcome. Models included county and year fixed effects and mean county-level age, sex, reason for Medicare entitlement, Medicaid dual-eligibility, and race and ethnicity (Asian, Black, Hispanic, White, and other [Native Hawaiian or other Pacific Islander]). To examine the possibility of ceiling effects, we estimated 2-way fixed-effects quantile regression models at the 25th, 50th, and 75th percentiles of study outcomes.^4^ Standard errors were bootstrapped and all results weighted based on MA enrollment. All analyses were performed using Stata version 17 (StataCorp).

## Results

Our analysis included 31 690 county-year observations from 2009-2019. Of the counties receiving double-bonuses, 80.5% became eligible in 2012, and 64.8% maintained eligibility during the entire postintervention period. In the pre-intervention period (2009-2011), double-bonus counties had higher performance than non–double bonus counties for both clinical measures (3.3 vs 3.0 stars) and administrative measures (3.6 vs 3.4 stars).

**Figure 2.**
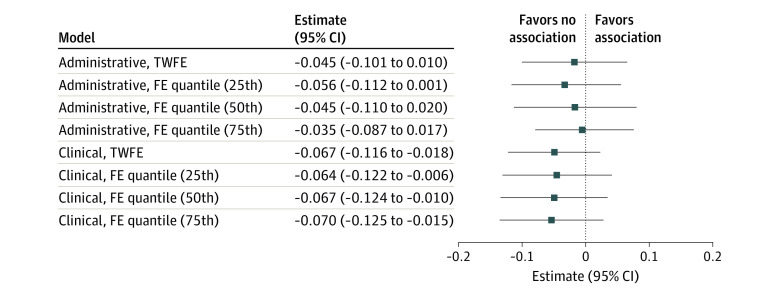
Association Between Double Bonuses and Clinical and Administrative Quality Squares denote point estimates and lines denote 95% confidence intervals. Quantile regression estimates capture the association between double bonuses and outcomes at the 25th, 50th, and 75th percentile of the distribution of the dependent variable. Standard errors for all models were estimated using a block bootstrap procedure with 1000 bootstrapping iterations. Standard errors for quantile models were estimated using de-meaned data. FE indicates fixed effects; TWFE, 2-way fixed effects.

Pre-intervention changes in the outcomes were approximately parallel between counties that ever received double-bonuses and non–double bonus counties (Figure 1). Double bonuses were associated with a small reduction in overall clinical performance (−0.067 stars; 95% CI, −0.116 to −0.018 stars) and administrative performance (−0.045 stars; 95% CI, −0.101 to 0.010 stars) (Figure 2). Estimates from quantile models were similar, suggesting that ceiling effects did not play a role in the lack of performance improvement associated with double bonuses (Figure 2).

## Discussion

In this study, double bonuses in MA were not associated with improvements in clinical or administrative plan performance, findings consistent with prior research.^5,6^ Our study is the first to our knowledge to show that double bonuses were not associated with improved administrative measures over which plans had more control. Because plans in double-bonus counties had higher initial performance, they reached 4-star performance (the threshold required for most bonuses) earlier, which may have attenuated subsequent incentives to improve. Limitations include use of plan-level data and assumptions that pre-intervention period differences in quality between double-bonus and non-double-bonus counties would have remained similar without double bonuses. Our results suggest that double bonuses in MA are a poor federal investment and should be eliminated.

## Notes

Supplement. **eMethods.** Clinical and Administrative Measures
