# Supplementary material for: Association Between Double Bonuses and Clinical and Administrative Performance in Medicare Advantage
Source: JAMA Health Forum. 2022 Sep 23;3(9):e223301. doi: 10.1001/jamahealthforum.2022.3301 (PMC9508651; doi:10.1001/jamahealthforum.2022.3301)
Supplement: Supplement. — eMethods. Clinical and Administrative Measures [file jamahealthforum-e223301-s001.pdf]

# Supplemental Online Content

Ryan AM, Gulseren B, Ayanian JZ, Markovitz AA, Meyers DJ, Brown EF. Association between double bonuses and clinical and administrative performance in Medicare Advantage. *JAMA Health Forum.* 2022;3(9):e223301. doi:10.1001/jamahealthforum.2022.3301

**eMethods.** Clinical and Administrative Measures

This supplemental material has been provided by the authors to give readers additional information about their work.

© 2022 Ryan AM et al. *JAMA Health Forum*.

**eMethods.** Clinical and Administrative Measures

For each year, we calculated plans' composite clinical (19-25 measures annually, 12 consistently targeted) and administrative performance (11-14 measures annually, 7 consistently targeted) by taking the mean star rating for all measures in each domain

Clinical measures include: Breast Cancer Screening; Colorectal Cancer Screening;* Annual Flu Vaccine;* Improving or Maintaining Physical Health;* Improving or Maintaining Mental Health;* Monitoring Physical Activity;* Adult Body-Mass Index (BMI) Assessment; Special Needs Plan (SNP) Care Management; Care for Older Adults-Medication Review; Care for Older Adults-Functional Status Assessment; Care for Older Adults-Pain Assessment; Osteoporosis Management in Women who had a Fracture;* Diabetes Care-Eye Exam;* Diabetes Care - Kidney Disease Monitoring;* Diabetes Care - Blood glucose Controlled;* Controlling Blood Pressure;* Rheumatoid Arthritis Management;* Reducing the Risk of Falling;* Improving Bladder Control; Medication Reconciliation Post-Discharge; Plan All-Cause Readmissions; Providing Certain Kinds of Care that Help Plan Members with Diabetes Stay Healthy; Statin Therapy for Patients with Cardiovascular Disease; Cardiovascular Care - Cholesterol Screening; Diabetes Care – Cholesterol Screening; Glaucoma Testing; Access to Primary Care Doctor Visits; Pneumonia Vaccine; Appropriate Monitoring for Patients Taking Long Term Medications; Osteoporosis Testing; Testing to Confirm Chronic Obstructive Pulmonary Disease; Antidepressant Medication Management (6 months); and Continuous Beta Blocker Treatment.

Administrative measures include: Getting Needed Care;* Getting Appointments and Care Quickly;* Customer Service;* Rating of Health Care Quality;* Rating of Health Plan;* Care Coordination; Complaints about the Health Plan; Members Choosing to Leave the Plan; Beneficiary Access and Performance Problems; Health Plan Quality Improvement; Plan Makes Timely Decisions about Appeals;* Reviewing Appeals Decisions;* Call Center - Foreign Language Interpreter and TTY Availability; Doctors who Communicate Well; Doctor Follow up for Depression; Follow up Visit after Hospital Stay for Mental Illness; Seriousness of Problems Medicare Found During an Audit of the Health Plan"; Enrollment Timeliness; Complaints Tracking Module; Accuracy of Information Members Get When They Call the Health Plan; and Time on Hold When Customer Calls Health Plan.
*Denotes a measures that was consistently targeted over the program

© 2022 Ryan AM et al. *JAMA Health Forum*.
